# Supplementary material for: A systematic review of the prevalence of lifetime experience with ‘conversion’ practices among sexual and gender minority populations
Source: PLoS One. 2023 Oct 4;18(10):e0291768. doi: 10.1371/journal.pone.0291768 (PMC10550144; doi:10.1371/journal.pone.0291768)
Supplement: S1 File — (DOCX) [file pone.0291768.s001.docx]

**Supplementary File 1.** Sample search strategy (MEDLINE)

MEDLINE Search January 2022

Database: Ovid MEDLINE(R) and Epub Ahead of Print, In-Process, In-Data-Review & Other Non-Indexed Citations, Daily and Versions(R) <1946 to January 03, 2022>

Search Strategy:

--------------------------------------------------------------------------------

1     "conversion therap*".mp. (314)

2     "conversion effort*".mp. (17)

3     "conversion practice*".mp. (11)

**4     or/1-3 (340)**

5     "“reparative therap*".mp. (137)

6     "“reparative effort*".mp. (8)

7     "“reparative practice*".mp. (2)

**8     or/5-7 (147)**

9     ("reorientation therap*" or "re orientation therap*").mp. (19)

10     ("sexual reorientation" or "sexual re orientation").mp. (17)

11     ("gender reorientation" or "gender re orientation").mp. (4)

12     ("gender identity reorientation" or "gender identity re orientation").mp. (0)

**13     or/9-12 (29)**

14     "sexual orientation change*".mp. (34)

15     "gender identity change*".mp. (14)

16     "gender expression change*".mp. (0)

**17     or/14-16 (46)**

18     "ex-gay".mp. (8)

19     "gender acceptance therap*".mp. (0)

20     ("reintegrative therap*" or "re integrative therap*").mp. (3)

21     "gay cure therap*".mp. (0)

22     (homosexual* adj5 cure).mp. (10)

23     "sexual identity fluidity exploration".mp. (0)

24     "psychological attempts to change a person’s gender identity from transgender to cisgender".mp. (1)

25     PACGI.mp. (4)

**26     or/18-25 (23)**

**27     4 or 8 or 13 or 17 or 26 (552)**

28     Bisexuality/ (4555)

29     exp homosexuality/ (32371)

30     exp "Sexual and Gender Minorities"/ (11276)

31     bisexual*.mp. (11840)

32     homosexual*.mp. (38002)

33     ("men who have sex with men" or msm).mp. (16377)

34     sexual orient*.mp. (7002)

35     "women who have sex with women".mp. (188)

36     "sexual minorit*".mp. (3624)

37     (gay* or lesbian*).mp. (15818)

38     (GLB* or LGB*).mp. (5918)

39     (queer* or two spirit*).mp. (1925)

40     (nonheterosexual* or "non heterosexual*").mp. (493)

41     Transgender Persons/ (4887)

42     transgender*.mp. (9637)

43     "gender divers*".mp. (1014)

44     "gender creativ*".mp. (13)

45     "gender minorit*".mp. (7715)

46     ((non-binary or nonbinary) and gender).mp. (654)

47     genderqueer*.mp. (108)

48     genderfluid.mp. (9)

49     "trans wom*".mp. (355)

50     "trans m*".mp. (5403)

51     "transwom*".mp. (274)

52     "transmen".mp. (160)

53     "gender affirm*".mp. (1379)

54     (mtf or ftm).mp. (3600)

55     (transfeminine or transmasculine).mp. (253)

56     ("sex* reassignment”" or "gender reassignment").mp. (1824)

57     ("gender identity disorder*" or GID).mp. (877)

58     (transex* or transsex* or trans sex*).mp. (4930)

59     Transsexualism/ (4116)

60     "gender dysphori*".mp. (1764)

**61     or/28-60 (76212)**

**62     27 and 61 (143)**
