# Supplementary figures and images for: A systematic review of the prevalence of lifetime experience with ‘conversion’ practices among sexual and gender minority populations
Source: PLoS One. 2023 Oct 4;18(10):e0291768. doi: 10.1371/journal.pone.0291768 (PMC10550144; doi:10.1371/journal.pone.0291768)

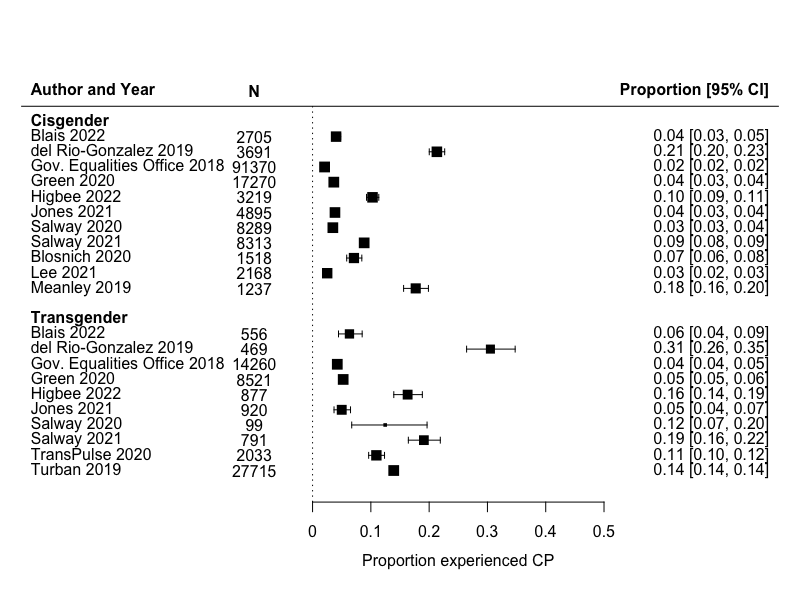

Supplement: S1 Fig — (TIF) [file pone.0291768.s003.tif]

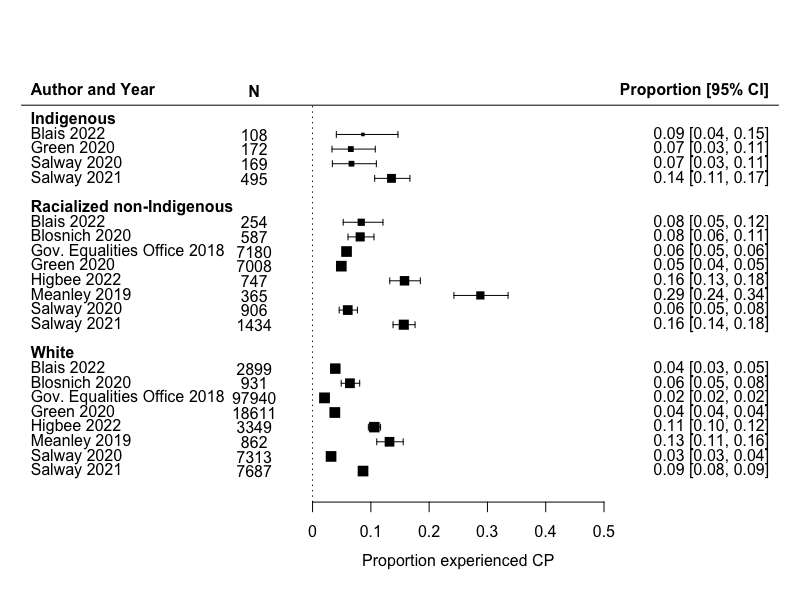

Supplement: S2 Fig — (TIF) [file pone.0291768.s004.tif]

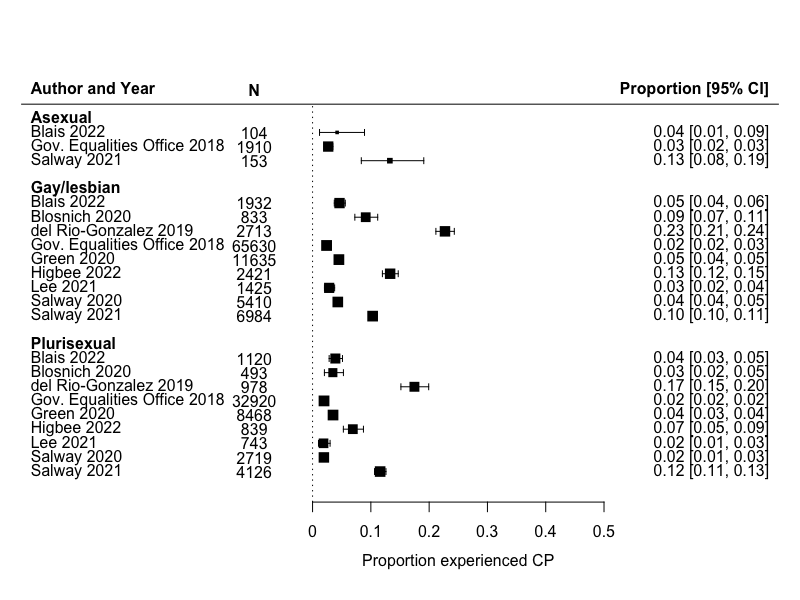

Supplement: S3 Fig — (TIF) [file pone.0291768.s005.tif]
