# Supplementary material for: A systematic review of the prevalence of lifetime experience with ‘conversion’ practices among sexual and gender minority populations
Source: PLoS One. 2023 Oct 4;18(10):e0291768. doi: 10.1371/journal.pone.0291768 (PMC10550144; doi:10.1371/journal.pone.0291768)
Supplement: Attachment — Submitted filename: PLoS ONE response to reviewers 20230719.docx [file pone.0291768.s007.docx]

Dear Dr. Baptista Silva,

We thank you and the reviewers for your thoughtful feedback on our manuscript, “A systematic review of the prevalence of lifetime experience with ‘conversion’ practices among sexual and gender minority populations.” We have edited the manuscript in response to these comments, as detailed below.

Sincerely,

Travis Salway & coauthors

| **Comment** | **Response** | **Edit (page number) in manuscript file** |
| --- | --- | --- |
| **Editor:** |  |  |
| Upon re-submitting your revised manuscript, please upload your study’s minimal underlying data set as either Supporting Information files or to a stable, public repository and include the relevant URLs, DOIs, or accession numbers within your revised cover letter. | We have uploaded the dataset as a .CSV file. | “The data used in this systematic review are available in a Supplementary File (“Dataset”).” (p28) |
| We note that you have provided funding information that is not currently declared in your Funding Statement. However, funding information should not appear in the Acknowledgments section or other areas of your manuscript. We will only publish funding information present in the Funding Statement section of the online submission form.  Please remove any funding-related text from the manuscript and let us know how you would like to update your Funding Statement. Currently, your Funding Statement reads as follows:  "TS, Canadian Institutes of Health Research (PCS - 168193)  https://cihr-irsc.gc.ca/  The funders had no role in study design, data collection and analysis, decision to publish, or preparation of the manuscript." | We have removed funding information from the Acknowledgments section of the manuscript.  Please update our Funding Statement as follows:  “This study was funded by the Canadian Institutes of Health Research (PCS – 168193). The funders had no role in study design, data collection and analysis, decision to publish, or preparation of the manuscript.” | n/a |
| **Reviewer 1:** |  |  |
| 1) In introduction part before to focus on CP practice please explore briefly the main problems that transgender people could copy in our society. For example in health care (Lavorgna L, Moccia M, Russo A, Palladino R, Riccio L, Lanzillo R, Brescia Morra V, Tedeschi G, Bonavita S. Health-care disparities stemming from sexual orientation of Italian patients with Multiple Sclerosis: A cross-sectional web-based study. Mult Scler Relat Disord. 2017 Apr;13:28-32. doi: 10.1016/j.msard.2017.02.001. Epub 2017 Feb 5. PMID: 28427697.) or from economic point of view. | We agree that we should contextualize CP with broader systemic factors (like cissexism) before describing the methodological issues in studying it. We have added a paragraph to the beginning of the manuscript, as recommended. | “CPs occur within a broader societal context of cissexism and heterosexism—belief systems that position cisgender and heterosexual identities and orientations as preferred and desirable.^1^ Cissexism and heterosexism lead to stigma, discrimination, violence, and a lack of protections and access to social determinants of good health for lesbian, gay, bisexual, transgender (trans), and queer (herein, sexual and gender minority [SGM]) people.^1^ This, in turn, produces numerous health disparities among SGM people, including unequal access to healthcare—a resource to which access in many settings is already limited by cost.^4^” (p4) |
| 2) please revise the English of the manuscript | Following reviewer 2’s comments regarding the Methods (specifically Analysis) section of the manuscript, we have added some clarifying language, which we hope improves the accessibility of the manuscript.  We are happy to make additional edits if the reviewer or editor have specific recommendations or requests. | See edits and parenthetical additions to Methods. (pp10-11) |
| 3) please add a graphical summary | We were unable to find instructions for graphical abstracts in the PLoS One submission guidelines so will defer to the editor. | n/a |
| **Reviewer 2:** |  |  |
| Here language is clear and attends to technical needs with no many difficult words. The introduction is very detailed and gives to the reader a complete description on the methods and shows the unique aspects and relevance of this paper, it does a really good job of giving a general idea to the whole text.  Something that could make the beginning a little more interesting for those who are reading would be to mention some data about gender and sexual diversity among the writers of the article. SGM people that are researchers and are reading the paper can create more boundaries to the research if they get to see diversity. | Thank you for the encouraging feedback regarding the Introduction.  We have added a brief author positionality statement to the first paragraph of the Methods section. | “We came to this study as an interdisciplinary group of health researchers invested in advancing equity- and evidence-informed public policy with SGM people, including in the context of CPs. Our authorship team includes SGM people and allies with expertise in population approaches to SGM health research.^1^” (p6) |
| The description of the methods is well structured. I really liked that it mentioned the use of references contemplating SGM writers, because this increases visibility and helps break up with stigma. | Thank you for the feedback. | n/a |
| Even though there are not relevant studies to the research that provides data about the economical aspects of SGM who experienced CP, this aspect could be a little more contextualized to the economical aspects of the countries in general. I´m pointing this out because a part of the SGM population, such as transgender people, a part of this community has needs on body modifications, to affirm gender and decrease dysphoria feelings some may proceed with surgeries, to put implants, to change the voice and other aspects... However, to access these procedures with safe medical circumstances guaranteed it's very expensive, at the same time, most of the countries do not offer the procedures in their public health systems. | We have added a paragraph to the start of the manuscript to contextualize CP, and we have included within this paragraph an acknowledgment of the cost-related barriers to gender-affirming care. | “CPs occur within a broader societal context of cissexism and heterosexism—belief systems that position cisgender and heterosexual identities and orientations as preferred and desirable.^1^ Cissexism and heterosexism lead to stigma, discrimination, violence, and a lack of protections and access to social determinants of good health for lesbian, gay, bisexual, transgender (trans), and queer (herein, sexual and gender minority [SGM]) people.^1^ This, in turn, produces numerous health disparities among SGM people, including unequal access to healthcare—a resource to which access in many settings is already limited by cost.^4^” (p4) |
| “Risk of bias”, is a concept that I never had heard of before reading this paper. This concept could be more detailed and explained, maybe it would be interesting to mention why the group chose Hoy´s method for this research. | We have added a definition of risk of bias, along with an explanation for our choice of the Hoy tool. There are few risk of bias tools available specifically for prevalence studies, and this one was the most flexible of those available. | “We assessed risk of bias among the studies included in the review in order to systematically describe methodological limitations to the available literature. We used the Hoy et al. risk of bias tool for prevalence studies, given its flexibility across research contexts.^26^” (p10) |
| This is the most complex part of the article. I understand the need of using all the scientific terms and concepts. However the writing here contains too many unusual words with a very specific vocabulary, this kind of language can become a challenge for researchers and readers that are not native english speakers. | Thank you for this feedback. We have added definitions and clarifying explanations to this section. | See edits and parenthetical additions to Methods. (pp10-11) |
| The way the authors present the results of this study is very satisfactory because it illustrates in many aspects the experiences that SGM faces during CP incidences. Sadly, the data presented reveals how oppressive and violent the manifestations of CP can be. One very positive aspect is the critical observation on how some of the articles do not define CP and how this can increase risk of bias and stigma between SGM groups. | Thank you for this feedback. | n/a |
| The discussion on the topic is one of the most important parts of this paper, because it is the part where the authors compare some features of the research based on the findings of the data analysis.  The limitations were well described and justified with the complexity of the subject. | Thank you for this feedback. | n/a |
| I suggest that in this topic, a more detailed analysis on the CP´s link to the religious groups or practices, and by doing so an intersectional relation with transgender community and gay man. It has been known that many religious centers can be responsible for creating violent processes to gender non-conforming groups and sexual minorities. Would be interesting to investigate the basis of the religions that are mentioned in these studies and point out who they are. If they are Catholic, Protestants, Jewish, or any other… | We have added a recommendation to this effect. | “Given that CP occur in both religious and secular contexts^8,29,35^, further research should investigate the degree to which particular religious ideologies continue to promote CP, as well as differences in the scope or nature of CP by religion.” (p26) |
